# Are quality assessments in science affected by anchoring effects? – Empirical results from a survey of authors assessing previously cited papers

**DOI:** 10.1371/journal.pone.0320148

**Published:** 2025-04-09

**Authors:** Lutz Bornmann, Christian Ganser

**Affiliations:** 1 Science Policy and Strategy Department, Administrative Headquarters of the Max Planck Society, Munich, Germany; 2 Department of Sociology, Ludwig-Maximilians-Universität Munich, Munich, Germany; Leiden University: Universiteit Leiden, NETHERLANDS, KINGDOM OF THE

## Abstract

Many studies have investigated anchoring effects. Anchoring occurs when initial values are used by humans as starting points in assessments. We investigated the prevalence of anchoring effects in the quality assessments of scientific papers. This study, which is preregistered, is a follow-up study that is intended to answer open questions from a previous study with the same topic. One open question concerns causal conclusions: it is necessary that randomly selected respondents assess the same paper under different conditions. In a survey, we asked corresponding authors to assess the quality of articles they have cited in previous papers. The respondents were randomly assigned to several experimental groups receiving numerical anchors such as citation counts or numerical access codes to the questionnaire. Although our results reveal scarcely effects of citation counts presented to the respondents as possible anchors, there is a small, but statistically significant effect of the random number (the numerical access code) presented to the respondents. Similar to other studies that have investigated the existence of anchoring effects in assessments in various contexts, our study could demonstrate the existence of an anchoring effect in research evaluation. Researchers seem to be influenced by numbers without any relationship to the quality of the evaluated paper in their assessment of papers.

## 1. Introduction

Tversky and Kahneman [1] call a phenomenon an anchoring and insufficient adjustment when initial values are used by humans as starting points in estimations: different numerical starting points lead to different numerical estimates. In order to demonstrate the effect of anchoring, Tversky and Kahneman [1] asked interviewed persons to estimate the percentage of African nations in the United Nations (UN). In the persons’ presence, a wheel of fortune was spun to receive a random number between 0 and 100. In the first instruction, the interviewed persons should indicate whether the random number is higher or lower than the African nations’ percentage “by moving upward or downward from the given number” [1]. In the second instruction, they were requested to estimate the percentage. The results of the experiment revealed a significant effect of the random numbers on the estimates: “For example, the median estimates of the percentage of African countries in the United Nations were 25 and 45 for groups that received 10 and 65, respectively, as starting points” [1].

Since the study of Tversky and Kahneman [1], many studies have investigated anchoring effects in various situations (of estimation, assessment or decision). Overviews of these studies can be found in Mussweiler, Englich and Strack [2], Furnham and Boo [3], Bahník, Englich and Strack [4], and Bahník, Mussweiler and Strack [5]. The literature overviews come to similar conclusions with respect to the prevalence of anchoring effects in various contexts. According to Mussweiler, Englich and Strack [2], for example, “anchoring effects are among the most robust and easily replicated findings in psychology” (p. 184). Furnham and Boo [3] concluded as follows: “research in the field demonstrates that anchoring is a pervasive and robust effect in human decisions” (p. 41). The authors of a meta-analysis [6] including studies that have investigated anchoring effects write: “anchoring has a significant impact on negotiators and negotiation outcomes” (p. 598). Previous research even demonstrated that anchoring effects appeared in situations in which persons were informed about the anchors, and instructions were used to correct anchoring effects.

Since assessments (under uncertainty) are prevalent in research evaluations, Bornmann, Ganser and Tekles [7] and Bornmann, Ganser and Tekles [8] studied for the first time whether anchoring effects also exist in the area of research assessments. The studying of anchoring effects is important, since quality assessments should be based on research itself, and not on (irrelevant) numbers which may bias the assessments. Following Traag and Waltman [9], we define bias as a direct causal effect of one variable on another variable that is unjustified (e.g., the effect of gender on scientific assessments). If scientific assessments are dependent on (irrelevant) anchors, the assessments are possibly biased and their validity can be questioned. The study of Bornmann, Ganser and Tekles [8] applied a similar study design as Teplitskiy, Duede [10] – although Teplitskiy, Duede [10] did not explicitly investigate anchoring effects. Bornmann, Ganser and Tekles [8] undertook a survey of corresponding authors from the Web of Science (WoS) database (Clarivate) whom they presented various information as possible anchors.

In the study of Bornmann, Ganser and Tekles [8], the corresponding authors were requested to assess the quality of articles that they cited in previous papers. The authors were randomly assigned to three treatment groups and a control group. The treatment groups received the following information alongside the title and abstract of the cited paper: paper impact information (measured by citations), impact information on the publishing journal (measured by citations), and a numerical access code to enter the survey. The control group did not receive any information besides title and abstract. Bornmann, Ganser and Tekles [8] explored on the one hand whether possible adjustments in the assessments of cited articles can be produced by information (paper impact or journal impact) that are related to quality. Their results reveal that the quality assessments of papers seem to depend on the paper, but not the journal impact information. The significant results on the paper impact information confirm similar results by Teplitskiy, Duede [10]. Bornmann, Ganser and Tekles [8] explored on the other hand the effect of numbers on quality assessments that are not related to quality, i.e., an access codes to the survey. With respect to the access codes, the results indicate that these arbitrary numbers do not play a role in the assessment of papers.

In this follow-up study, we investigated open questions from the results of Bornmann, Ganser and Tekles [8]. One open question refers to the paper impact information that revealed a statistically significant effect in the study by Bornmann, Ganser and Tekles [8]. The effect may be interpreted as a confirmation of the paper impact information as anchor whereby quality adjustments were caused by anchors. The results of Bornmann, Ganser and Tekles [8] yet do not allow this causal conclusion, since the results are based on a limited design: the respondents in the groups (receiving paper impact information or not) assessed different cited articles. Not only the paper impact information was varied in the study, but also the assessed article (and, by association, the article’s quality may have also varied). For causal conclusions, it is necessary that randomly selected respondents assess the same cited paper under two conditions: with (different) or without paper impact information.

Another open question of the study by Bornmann, Ganser and Tekles [8] concerns the investigated influence of the access code on the quality assessments. The missing effect of the access code in the study may result from the handling of the access code in the questionnaire. Since Bornmann, Ganser and Tekles [8] only requested the respondents to fill in the access code in the questionnaire, respondents were not demanded to engage more deeply with the access code. We tried to stimulate this engagement in the follow-up study. Following the classical study by Tversky and Kahneman [1], the respondents assessed in a first step whether the quality assessment is higher or lower than the access code. In the second step then, the absolute quality assessments followed – as was done by Bornmann, Ganser and Tekles [8]. We expected that the engagement of the respondents with the access code is intensified by asking for the relative assessment in the first step, and an effect is possibly evoked on the absolute quality assessment in the second step. This stimulation of the engagement has been also done for the citation counts presented to the respondents (to have the same experimental conditions for all respondents).

## 2. Literature overview

This literature overview is mainly based on the overviews by Mussweiler, Englich and Strack [2], Bahník, Englich and Strack [4], and Bahník, Mussweiler and Strack [5]. Anchoring can be defined as “the assimilation of a numeric judgement to a previously considered standard“ [2]. An anchoring effect has been detected in different judgement situations (e.g., knowledge questions, price estimates, and legal judgements). Anchoring effects have been demonstrated in absolute judgments (e.g., how great is something) and in comparative judgments (e.g., is something smaller or larger). Anchors may be of different type: they can be numeric values or specific text stimuli. Effects of anchors have been generated in laboratory settings [e.g., 1] and in ‘real-world’ settings [e.g., 11].

Anchoring effects have been investigated in the context of four experimental paradigms, in which “the anchor values are either explicitly or implicitly provided by the experimenter, self-generated, or provided in an unrelated task” [2]. The paradigm in many studies is the approach by Tversky and Kahneman [1], where the anchor values are provided in an unrelated task including first comparative and then absolute anchoring questions. This study is oriented towards this usual approach.

Studies in the four experimental paradigms have revealed that anchoring effects are robust with respect to various moderating variables. Anchors can be differentiated whether or not they are relevant for a judgmental task to be effective. In the study by Bornmann, Ganser and Tekles [8], e.g., citation information can be interpreted as relevant for quality assessments of papers, but access codes as not relevant. One may assume that only relevant anchors are effective in assessments. Furnham and Boo [3] concluded yet in their overview of the literature that “irrelevant anchors produce similar effects in judgmental decisions in comparison to those of informational relevance anchors” (p. 38). Research also demonstrated that anchoring effects are effective in situations in which humans tried to work against the influence of presented anchors (e.g., by using instructions in the study to correct for an influence of the anchor). Extremity and implausibility of anchors also do not seem to prevent anchoring of being effective.

Five theoretical backgrounds have been proposed for explaining anchoring effects: it has been suggested that “anchoring effects result from (1) insufficient adjustment from an anchor, (2) conversational inferences, (3) numerical priming, (4) mechanisms of selective accessibility, and (5) distortion of the response scale“ [5].

## 3. Citation counts as possible anchors in quality assessments

Doing science is not imaginable without research evaluation: “scientific progress is nowadays strongly dependent on research evaluation processes, as they regulate the stream of ideas and research projects by means of science funding allocation” [12]. The use of citations for research evaluation has a long tradition: the term evaluative bibliometrics was introduced by Narin [13] decades ago based on the structural-functional approach by Merton [14]. Citation analyses are (currently) used for the evaluation of research groups, institutions, and countries as well as of research proposals and hiring of academic personnel [15]. For example, Sivertsen [16] published an overview of the use of various bibliometric indicators in national performance based research funding systems of several countries such as Italy and the United Kingdom (UK). Bibliometrics has this prominent role in research evaluation, since it combines four advantages [17]: (1) bibliometrics is able to provide single numbers that may reflect research performance; (2) bibliometrics may provide performance numbers that are comparable across disciplines (by the use of field-normalized indicators); (3) bibliometrics can be used unobtrusively with available process-oriented numbers from large databases; (4) the costs of using bibliometrics are (usually) low when compared with the use of peer review panels.

The frequent use of bibliometrics in research evaluation is accompanied by massive critique – despite its many advantages. An overview of important critical points can be found in Jappe, Pithan and Heinze [18]. In this study, we address one of these points: does bibliometrics create the social order in science it is designed to measure? If so, the social order may be independent from the intrinsic quality of research. The opposite assumption is that bibliometrics reflects the given social order (which is dependent on the intrinsic quality of research). If bibliometrics creates the social order, it can be interpreted as an anchoring-and-adjustment heuristic [1]. Bibliometrics would then mean a starting point of an evaluator’s assessment about a certain piece of research.

## 4. Data and methods

The datasets and methods used are explained in detail in the preregistration of this study [19]. The preregistration also explains possible limitations.

### 4.1. Dataset used

Between December 2023 and March 2024, we undertook a survey of corresponding authors with an available email address in the Web of Science database (in-house database at the Max Planck Society, Germany). We informed the corresponding authors of the purpose of the study, and since there was no harm associated with participation, explicit consent was not considered necessary. Participants who did not wish to share their opinions could simply ignore the invitation e-mail. Our entire study design, including the content of the e-mail and the questionnaire, was thoroughly reviewed and approved by the Research Ethics Committee of the Faculty of Social Sciences at the Ludwig-Maximilians-Universität Munich. This approval, together with the documents on which the ethics committee based its decision, was included in the pre-registration of the study [19]. Neither the ethics committee nor the reviewers raised any ethical concerns about the study.

The corresponding authors received an email with a link to a web-based questionnaire with the request to assess the quality of a paper that they cited some years ago. They were randomly assigned to one of five author groups which received (or not):

1. Information about the (true) citation impact of the cited paper as an anchor with possibly relevant information for the quality assessment (the author was informed about the use of the citation impact as possible anchor in this study in the invitation email),
2. Information about the (true) citation impact of the cited paper (the author was not informed about the use of the citation impact as anchor),
3. A randomly generated anchor (an access code to the questionnaire) that is not related to the quality of publications (the author was informed about the use of the access code as anchor) or
4. A randomly generated anchor (an access code to the questionnaire; the author was not informed about the use of the access code as anchor).
5. A fifth group of authors did not receive any additional information that may function as anchor.

Respondents were first asked whether they remembered the presented paper (previously cited). If not, up to three alternative cited papers were presented to them. Further questions referred to how well they knew the paper which was assessed, and how much it influenced the citing paper (their own paper). In the most important part of the questionnaire, the respondents were first asked whether the quality assessment is higher or lower than the presented access code or citation count, respectively. Second, the respondents rated the previously cited paper with respect to several characteristics: overall quality, novelty (i.e., departed significantly from what is usually done in this research area), significance (i.e., addressed an important topic), validity (i.e., designed and performed very well, produced very credible results), generalizability (i.e., easily applicable to other contexts), and canonical or prominent reference [i.e., the standard reference for this topic; the terms canonical and prominent were randomized, following [10]. The range of the rating scale was from 1 (bad) to 100 (excellent).

We included in our study cited papers from 2010 that belong to the 1% most frequently cited papers in their publication year and subject category. The main reason for the decision to include older papers with many citations was to make sure that we receive multiple ratings for each paper (since many cited papers and citing authors exist). The range of the quality assessment scores is from 1 to 100. The citation counts and access codes that were used in the study are nearly in the same range. Many highly cited papers in our sample have high citation counts, i.e., higher than the range of possible quality assessment scores. Some highly cited papers have no or only one citation in the first three (five) years after publication. To prevent the possibility that the citation counts are out of the range (with respect to the corresponding quality assessment scores), we decided not to consider cited papers in the planned study with citation counts lower than 2 and higher than 99.

### 4.2. Sample

In total, 12,041 participants answered our questionnaire and provided assessments of the quality of the presented (previously cited) papers. This corresponds to a response rate of 2.84%, which seems to be low but it is in the range we expected from Bornmann, Ganser and Tekles [8]. 4,704 papers have been assessed by the respondents. On average, each paper received 2.6 ratings but the distribution of ratings is skewed. While the maximum of ratings per paper is 36, 66.2% of the papers received only 1 or 2 ratings. 1,339 papers received between 3 and 6 ratings (see Table 1 for details).

**Table 1:** Number of ratings per paper.

Number of ratings	Papers (absolute)	Papers (in percent)
1	1,959	41.7
2	1,152	24.5
3	622	13.2
4	361	7.7
5	238	5.1
6	118	2.5
7	87	1.9
8	40	0.9
9	26	0.6
10+	101	2.2
Total	4,704	100.0

The respondents are almost evenly distributed over the five experimental groups (see Table 2). This indicates that the randomization has worked.

**Table 2:** Distribution of respondents.

Experimental group	Respondents (absolute)	Respondents (in percent)
Citation count, revealed as anchor	2,254	18.72
Citation count, not revealed as anchor	2,447	20.32
Access code, revealed as anchor	2,207	18.33
Access code, not revealed as anchor	2,593	21.53
Control group	2,540	21.09
Total	12,041	100.0

### 4.3. Statistics applied

We computed multilevel models in this study, because we have repeated measures for the cited paper in our dataset. We examined treatment effect heterogeneity by estimating fixed effects regression models, including the values of the treatments. This allows us to compare effect sizes of the treatments. The model is specified as


$$\begin{array}{l} quality\;assessment_{ij}\\ \;\;\;\;\;\;=\beta_{0}+\beta_{1}condition2_{ij}+\beta_{2}condition3_{ij}+\beta_{3}condition4_{ij\;}\\ \;\;\;\;\;\;+\beta_{4}condition1\cdot value+\beta_{5}condition2\cdot value\\ \;\;\;\;\;\;+\beta_{6}condition3\cdot value+\beta_{7}condition4\cdot value+u_{j}+e_{ij} \end{array}$$

where *β*_*0*_ is the intercept, *β*_*1*_ to *β*_*7*_ are the coefficients of the respective independent variables, “condition” 1–4 are the four treatment conditions (see above), “value” is the numerical value of the respective information presented to the respondents, *u*_*j*_ is an error term on the paper level, and *e*_*ij*_ is an error term on the observation level.

To facilitate the interpretation of the results, this model does not include a main effect for “value” but four interactions. This also allows us to include the citation counts in the models, because the interactions with the treatment conditions are not constant for a given paper. The coefficients of the interaction terms represent the effects of the “value” on the quality assessments for the four treatments. We refer to these interactions in Section 5, because the main effects of the conditions are not the foci of our research questions.

Additionally, we estimated a pooled OLS regression model. The model does not include fixed effects for the papers, i.e., it does not control for the quality of the papers. This additional analysis allows us to investigate how controlling of the papers’ quality in the fixed effects regression models affects their results.

## 5. Results

### 5.1. Descriptive results

Table 3 shows descriptive statistics of the seven outcome variables (the various aspects of quality) and two independent variables used in the results section (see Section 5.2) and in several robustness checks (see Section 5.3). All quality variables in the table are left skewed, i.e., the papers are rated better than a typical paper for the respective field. This is not surprising because we selected highly cited papers for our sample. Highly cited papers are expected to be rated higher. The results in Table 3 reveal that there is enough variation in the ratings to use them as dependent variables.

**Table 3:** Descriptive statistics.

	Mean	Median	Standard deviation	Skewness	*N*
Dependent variable (1–100)					
Overall quality	71.20	75.00	16.87	-0.74	12,041
Novelty	66.80	70.00	19.05	-0.44	11,394
Significance	74.35	77.00	17.22	-0.84	11,832
Validity	75.27	80.00	17.91	-0.99	11,038
Generalizability	70.41	75.00	19.99	-0.68	11,197
Canonical	68.78	71.00	22.15	-0.60	5,538
Prominent	70.05	75.00	21.79	-0.73	5,699
Independent variable					
Citation count	54.83	55.00	24.93	-.06	4,698
Proximity (range 1–100)	36.46	26.00	30.98	0.52	11,687
Knowledge (ordinal, 1–5)	(3.14)	3.00	(1.10)	(0.08)	12,000

Notes. Access codes are uniformly distributed by design in the interval from 1 to 99

The proximity of the respondents’ own areas of research to the research reported in the cited paper is also skewed (see Table 3): More papers are close to the authors’ own field of research, which also was to be expected. The results in the table reveal that the distribution of the knowledge of the cited paper is almost symmetrical. Most respondents reported an average knowledge of the paper assessed.

### 5.2. Effects of presented values on quality assessments

**Fig 1:**
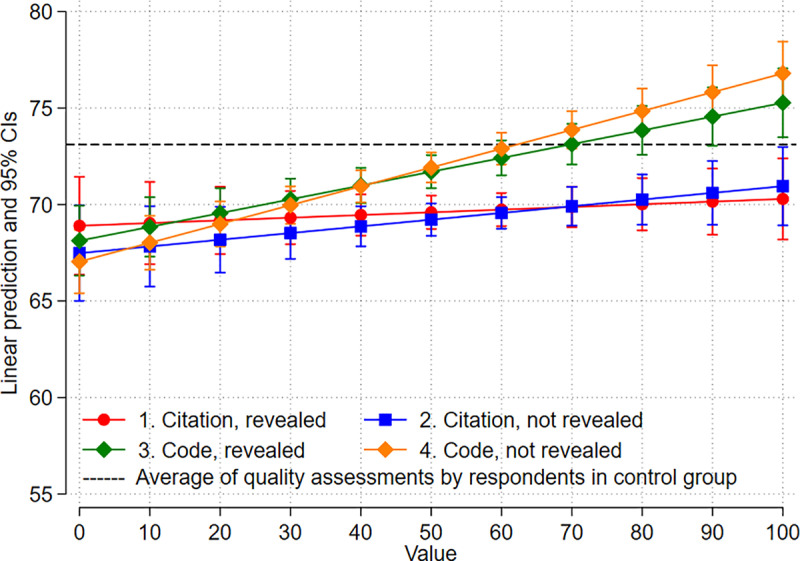
Predicted quality assessments depending on presented numbers (adjusted predictions with 95% confidence intervals, CI). The control group assessed the papers without number presentations.

Table 4 shows the results of the regression analysis investigating the relationship of overall quality assessments and numbers presented to the respondents for the four groups in our survey (e.g., citations). Fig 1 reveals predicted values (with 95% confidence intervals, CIs) of the overall quality assessments depending on the number presented to the respondents for the four groups based on the regression results in Table 4. The average of the quality assessments by the respondents in the control group, who assessed the papers without number presentations, is additionally included in the figure for comparison.

**Table 4:** Fixed effects regression of quality assessments on presented numbers.

Variable	Coefficient
Citation, not revealed	−1.420
	(−0.92)
Code, revealed	−0.775
	(−0.48)
Code, not revealed	−1.858
	(−1.20)
Citation, revealed x value	0.0139
	(0.63)
Citation, not revealed x value	0.0347
	(1.61)
Code, revealed x value	0.0714^***^
	(4.42)
Code, not revealed x value	0.0975^***^
	(6.60)
Constant	68.90^***^
	(53.23)
*N* (observations)	9,498
*N* (papers)	4,223
*R* ^2^	0.017

Notes. *t* statistics in parentheses; *  *p* < 0.05, ** *p* < 0.01,

***p < 0.001

The results of Table 4 and Fig 1 demonstrate that there is a small, yet statistically highly significant (*p* < .001) positive effect of the access code presented to the respondents on the overall quality assessment. An increase in the access code of ten points (on a scale from 1 to 99) leads to an increase of the assessments by 1 point if the usage of the code as an anchor is not revealed to the respondent. The access code leads to an increase of 0.7 points if the usage as an anchor is revealed. Citation counts show no statistically significant effects, regardless of whether their use as an anchor was disclosed or not.

**Fig 2:**
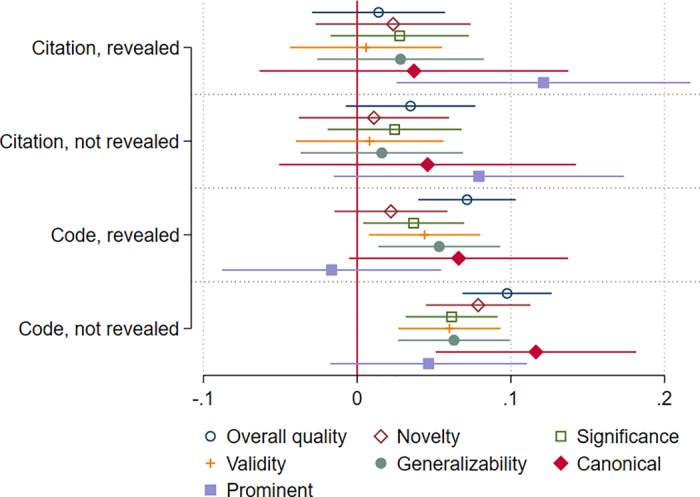
Effects of presented numbers on assessments of various aspects of quality.

For the detailed quality aspects (“novelty”, “significance”, “validity”, “generalizability”, and “canonical reference”), the pattern substantially hold what we found for the overall quality. Table A6 in the S1 Appendix document presents the results of the regression analyses and Fig 2 the predicted values based on these analyses. Table A6 and Fig 2 demonstrate that citation counts revealed as an anchor to the respondents does not have statistically significant effects except for “prominent”. When the citation counts’ anchor use was not revealed, citation counts have no statistically significant effects. For the access code revealed as an anchor, there is a statistically significant effect on four of the seven outcome variables (“overall quality”, “significance”, “validity”, and “generalizability”). The effects on the assessments of “significance” and “validity” are statistically significant at the 0.05 level, while the *p*-value of the effect on the assessment of “generalizability” is 0.008.

Access codes that were not revealed as anchors have statistically significant effects on the assessments of all aspects of quality except for “prominent”. For “generalizability”, the *p*-value is 0.001, for the other aspects the effects are statistically highly significant (*p* <  0.001). Since “canonical” and “prominent” were presented only to one half of the respondents each, the *p* values should not be compared with the other quality aspects. The found effect sizes are small in all models that we estimated. Most of the effects are below 0.1, meaning that an increase in the independent variable of 10 points leads to an increase of the quality assessments of less than one point.

It might be surprising that the access codes have stronger effects on the quality assessments than the citation counts, because the latter are assumed to correlate at least to some degree with the quality of papers. However, the result may no longer be surprising, if one considers in the interpretation that the quality of the papers is controlled by the fixed effects models computed in this study. The results seem to confirm the relationship of citation counts and quality. Otherwise, we would have observed an “additional” effect of the citation counts in the results.

This interpretation is underpinned by a pooled OLS regression model that we estimated additionally (this model does not control for the quality of the papers, see Table 5). In this model, citation counts have an effect of 0.028 – which is statistically significant at the 0.05 level (*p* =  0.045) – when their use as an anchor is revealed to the respondents. A highly statistically significant (*p* =  0.000) effect of 0.05 is shown when their use as an anchor is not disclosed. Access codes have statistically highly significant effects in this model as well (*β* =  0.063, *p* =  0.000 when revealed as an anchor; *β* =  0.078, *p* =  0.000 when not revealed). This means that the effect of the citation counts is reduced by 50.9% (revealed as anchor) and 30.3% (not revealed) and not statistically significant anymore, while the effect of the randomly generated anchors remains statistically significant and even gets stronger in the fixed effects models compared to the pooled model.

**Table 5:** Pooled OLS regression of quality assessments on presented numbers.

Variable	Coefficient
Citation, not revealed	−1.626
	(−1.39)
Code, revealed	−0.116
	(−0.10)
Code, not revealed	−0.514
	(−0.48)
Citation, revealed x value	0.0283^*^
	(2.00)
Citation, not revealed x value	0.0498^***^
	(3.71)
Code, revealed x value	0.0634^***^
	(5.08)
Code, not revealed x value	0.0783^***^
	(6.78)
Constant	68.39^***^
	(80.68)
*N* (observations)	9,498
*N* (papers)	4,223
*R* ^2^	0.013

Notes. *t* statistics in parentheses;

*p < 0.05, ** *p* < 0.01,

***p < 0.001

### 5.3. Robustness

In this section, we describe the results of some robustness checks that we have performed in addition to the results in Section 5.2. The checks refer to the regression model with the overall quality as dependent variable.

#### 5.3.1. Reviews and technical reports.

While we selected only papers of the type “article” for our sample, some respondents alerted us that we asked them to rate review articles or technical reports. Since the WoS database uses other document type categorizations than publishers, such feedback from respondents is understandable. Reviews and technical reports are usually cited very frequently but for other reasons than original research articles (e.g., reviews give an overview of the research in a field or on a topic). The different motives to cite reviews and technical reports might bias our results. Our results show, however, that the exclusion of 192 articles with one or more of the terms “review”, “overview”, “meta-analysis”, “technical”, and “report” in the title of the papers does not alter the results substantially (see Table A7 in the S1 Appendix document).

#### 5.3.2. Citation numbers.

In the survey, we presented citation counts from the in-house database of the Max Planck Society to the respondents from the first 3 or 5, respectively, years after the appearance of the cited publication. Respondents might have doubted the presented citations counts (e.g., they may have had the impression that the number is too low). In the end of the questionnaire, therefore, we asked the respondents whether they searched for the number of citations themselves. Excluding the 979 respondents who did not answer this question with “no” (these respondents could have searched for the citation counts) did not alter our results substantially (see Table A8 in the S1 Appendix document). Furthermore, we excluded in another analysis the ratings for the 95 cited papers, where the citation counts up to 2021 are more than 20 times higher than the citation counts presented in the survey (the presented low numbers may have irritated the respondents). This exclusion also had no substantial effect on the results of the regression analysis (see Table A9 in the S1 Appendix document).

#### 5.3.3. Field of citing paper.

Depending on the subject category, there are varying publication and citation cultures in science [20]. We expected, therefore, that the importance attributed to citation counts for the assessment of the quality of a paper might also vary between fields. To address this issue, we conducted separate regression analyses for the subject categories of the citing papers. We distinguished the following fields: natural sciences, engineering and technology, medical and health sciences, agricultural sciences, social sciences, and humanities. For agricultural sciences and humanities, there were too few respondents to conduct separate analyses (311 and 195, respectively). The results of the models for the remaining fields underpinned the main results (see Table A10 in the S1 Appendix document). Citation counts have no statistically significant effects on the quality assessments with only one exception: In the social sciences, citation counts – not revealed to the respondents as an anchor – have a positive effect on the quality assessments. The effect is statistically significant on the 0.05 level (*β* =  0.102, *p* =  0.028). In four out of eight cases, citation counts showed positive and statistically significant effects on the quality assessments. In the natural sciences, access codes have an effect of *β* =  0.092 when their use as an anchor is revealed to the respondents and of *β* =  0.090 when it is not. Both effects are highly significant (*p* =  0.000). In engineering and technology, the access codes have an effect of *β* =  0.123 (*p* =  0.049) if their use as an anchor is revealed; the effect is statistically not significant when the use as an anchor remains undisclosed. In the medical and health sciences we also found the expected pattern: The effect of the random access codes is stronger when their use as an anchor was not revealed (*β* =  0.140, *p* =  0.000) compared to the condition when their use was revealed (*β* =  0.029, *p* =  0.484).

#### 5.3.4. Knowledge of cited paper and distance to own field.

Since individuals tend to base their decision stronger on heuristics when they have fewer information, one can expect that anchoring effects occur especially when respondents have less knowledge of the paper they assess. We asked respondents to rate their knowledge of the cited paper on a scale from 1 (“Extremely well (know it as well as my own work)”) to 5 (“Not well (only familiar with main findings)”). Interactions of this scale (treated as a continuous variable) with the values of the treatments show that the effects of the four treatments do not depend on the knowledge of the assessed papers. All interaction effects are not statistically significant (see Table A11 in the S1 Appendix document).

**Fig 3:**
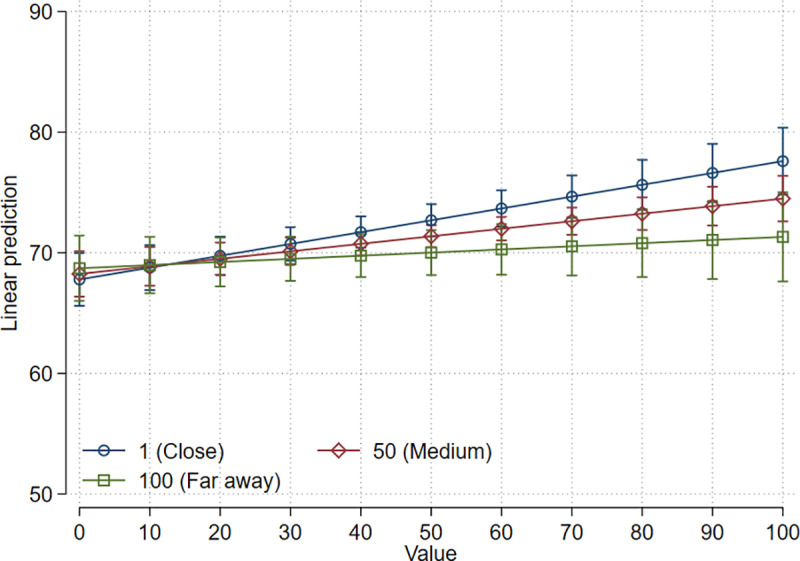
Effects of presented numbers on assessments of various aspects of quality.

Following Mussweiler, Englich and Strack [2], we assumed that the distance between the own field of research and the field in which the rated paper was published could alter the intensity of anchoring effects. The more familiar a researcher is with the field a paper stems from, the easier it should be to assess its quality and therefore researchers should be less prone to anchoring. In our survey, respondents were asked to indicate the proximity of their own area of research to the research reported in the cited paper on a scale from 1 (very close) to 100 (far away). Interaction effects of this scale with the treatment values were not statistically significant with the expectation of access codes, revealed as an anchor (see Table A12 in the S1 Appendix document). However, the effect we found is in the opposite direction than what we expected. Since the coefficient is negative (-0.0007, *p* =  0.041), anchoring effects are weaker the greater the distance is. If the proximity is 1, the effect of the code is 0.098, if it is 100, the effect declines to 0.026 (see Fig 3).

## 6. Discussion

Imagine we asked you if the average temperature in Paris was higher or lower than 35 degrees Celsius. Do you think this would affect your estimate of the city’s average temperature in comparison to a similar question without a reference value? Now, consider if we asked whether the average price of a college textbook was higher or lower than €76. Would this question influence your estimate of the textbook’s average price? Scientists (in psychology) refer to such effects as “anchoring.” The initial figure we encounter often serves as a reference point, unduly influencing our expectations about the actual value of an item [6]. One of the most consistent findings in understanding how people make numeric judgments is derived from anchoring research: Individuals tend to use recently encountered numbers as a basis for their judgments. Unlike a normative decision-making process, where people would methodically weigh informational inputs, thoroughly search their memory for relevant data, and ignore irrelevant numbers, numerous studies indicate that people often incorporate clearly uninformative numbers encountered before making a decision. The anchoring effect – where judgments are influenced by numbers present in the environment – has exemplified bias in human judgments for the past fifty years [21]. The results of a meta-analysis including the extensive literature on anchoring containing 2,131 total effect sizes found “a large (*d* = 0.876, 95% *CI*[0.808, 0.943], *I*^*2*^ = 92.96%) effect with only a small reduction from publication-bias corrections” [21].

In this study, we investigated the prevalence of anchoring effects in the quality assessments of papers. Our results show that there is a small, but statistically significant effect of a random number (access code) presented to the respondents. This effect was stronger when the respondents did not know that the random number is used as an anchor. When this anchor was revealed, there were fewer statistically significant effects on the assessments of various quality aspects of the cited (presented) paper and the effect sizes were even smaller. There were scarcely effects of citation counts presented to the respondents as possible anchors, regardless of whether the respondents were informed about their use as an anchor or not. The missing effects of citation counts (compared to the effects of the random number) seem surprising because citations are seen as at least weakly correlated with the quality of papers [see, e.g., 22]. Since the quality of the papers was controlled in the analyses by using fixed effects models, missing effects can still be expected: In the statistical analyses, only the interactions of the within variation of the numerical value – that were presented to the respondents with the respective condition – were considered. The results seem thus to confirm the relationship of citation counts and quality. Otherwise we would have observed in the results an “additional” effect of the citation counts.

Similar to other studies that have investigated the existence of anchoring effects in human assessments, our study – with an optimized design of the previous study by Bornmann, Ganser and Tekles [8] – could demonstrate the existence of an anchoring effect in research evaluation. Researchers seem to be influenced by numbers – i.e., numbers without any relationship to the quality of the evaluated paper – in their assessment of papers (but the effect of the influence is small). Previous research also has demonstrated that anchors can influence estimates even when they are unrelated to the target question. For instance, in a study by Wilson, Houston and Brekke [23], participants were asked to copy an ID number (serving as the anchor) before estimating the number of physicians listed in a phone book. Despite the lack of direct connection between the anchor and the question, the results reveal a substantial influence: participants who copied higher ID numbers estimated a greater number of physicians in the phone book [24]. Furnham and Boo [3] summarize the literature on irrelevant anchors as follows: “In short, irrelevant anchors produce similar effects in judgmental decisions in comparison to those of informational relevance anchors”.

Anchoring does not explain the underlying mechanisms; it merely indicates the direction of the observed effect, specifically the tendency for final judgments to align with an initial value: “the term ‘anchoring’ constitutes a descriptive rather than an explanatory concept which does not go beyond the terms ‘assimilation’ and ‘contrast’” [25]. There is still an ongoing discussion about the processes that may be active behind anchoring. In the following, we take up existing theories about the mechanisms behind anchoring and discuss the theories against the backdrop of the results of this study and the precursor study [8]. The studies found that (1) citations are used as anchors by the respondents but seem to reflect the quality of papers. The quality assessments of the respondents are related to the numbers of citations. (2) Irrelevant numbers (such as an access code to a questionnaire) are able to serve as anchors in quality assessments of papers.

First, it has been proposed that anchoring is an effect that results from conversational inferences: “In certain situations, an anchor can influence judgment because it is perceived as an informative cue to a given task” [26]. Conversational inferences may explain the effect of citations on quality assessments since the respondents assess citations as informative cues. However, conversational inferences cannot explain the effect of access codes on the quality assessments, since codes and assessments are not related in any way. 

Second, another theory of anchoring concerns an insufficient adjustment of the respondent from the anchor value: “The anchoring effect could also occur because people insufficiently adjust their judgment from an anchor … The insufficient adjustment may happen because it is cognitively effortful” [26]. This explanation of anchoring may be relevant for both effects we found. It may be cognitively effortful for respondents to adjust their assessments not only from citations but also from access codes. In case of citations, adjustments may not be necessary since their consideration may improve the quality assessments of the respondents.

Third, anchoring may be the result of selective accessibility of information that is compatible with an anchor: “consideration of the anchor activates information compatible with the possibility that the target value is equal to the anchor value. This activated information is thus more accessible and therefore more likely to be used in a subsequent judgment” [26]. This theory may be relevant for both anchors used in this study: citations and access codes. Both numbers may lead to the search by respondents for information that may be congruent with the presented anchor value (e.g., information on the reputation of the cited papers’ authors or its publishing journal). 

Fourth, anchoring may result from a distortion of a response scale: “anchoring affects people’s perceptions of the response scale without affecting their perception of the target object itself” [26]. The fourth explanation focuses only on the response scale but not on the assessed object: respondents may have a certain opinion about the quality of a cited paper, and this opinion does not change although the citations’ or access codes’ anchors are effective. The reason is – following this theory – that the anchor values only affect the responses on the given scale (we used the same scales for the anchor values and the quality assessments) but not the assessment of (the opinion about) the given paper.

We can imagine that the four explanations for anchoring are relevant for the explanation of the effects of citations and access codes on scientists’ assessments of papers. Since the extent of their relevance is not clear yet, we recommend that forthcoming research investigates the various theories for anchoring. Research questions may be as follows: Do the respondents activate some information about a cited paper (that is presented in a survey) to adjust the quality assessment to a certain value (that is also presented)? If so, it would be interesting to explore which information are activated and how they are related to the quality of the cited paper. Another study may focus on the question whether anchoring “only” concerns the response scale of the quality assessments or influences (also) the opinion about the quality of the cited paper. Is it imaginable that citation counts have “a life of their own” (and can be influenced by anchors) but the opinion about certain papers is not influenced by a separated “world of bibliometric indicators”? Since the four theories on anchoring can be translated into many research questions in the area of research evaluation, our questions are only a few examples. Further ideas for other research questions can be derived from the anchoring research in other areas than research evaluation.

## Supporting information

S1 Appendix Containing Table A6 to Table A12. (DOCX)
