# Supplementary material for: Are quality assessments in science affected by anchoring effects? – Empirical results from a survey of authors assessing previously cited papers
Source: PLoS One. 2025 Apr 9;20(4):e0320148. doi: 10.1371/journal.pone.0320148 (PMC11981202; doi:10.1371/journal.pone.0320148)
Supplement: S1 Appendix — (DOCX) [file pone.0320148.s001.docx]

S1 Appendix

Are quality assessments in science affected by anchoring effects?

Empirical results from a survey of authors assessing previously cited papers

Lutz Bornmann* & Christian Ganser+

* Science Policy and Strategy Department

Administrative Headquarters of the Max Planck Society

Hofgartenstr. 8,

80539 Munich, Germany.

Email: bornmann@gv.mpg.de

+ Ludwig-Maximilians-Universität Munich

Department of Sociology

Konradstr. 6

80801 Munich, Germany.

Email: christian.ganser@soziologie.uni-muenchen.de

Table A6. Fixed effects regression of various quality aspects on presented numbers.

| Variable | Overall quality | Novelty | Sig­nificance | Validity | Generali­zability | Canoni­cal | Pro­minent |
| --- | --- | --- | --- | --- | --- | --- | --- |
| Citation, not | -1.420 | -0.828 | -0.784 | -1.406 | 0.613 | -0.541 | 1.246 |
| revealed | (-0.92) | (-0.46) | (-0.49) | (-0.80) | (0.32) | (-0.15) | (0.36) |
|  |  |  |  |  |  |  |  |
| Code, | -0.775 | -0.684 | 0.308 | -2.431 | -0.195 | 0.686 | 10.16^**^ |
| revealed | (-0.48) | (-0.36) | (0.18) | (-1.32) | (-0.10) | (0.19) | (2.79) |
|  |  |  |  |  |  |  |  |
| Code, not | -1.858 | -2.934 | -0.320 | -2.372 | -0.219 | -0.574 | 7.192^*^ |
| revealed | (-1.20) | (-1.63) | (-0.20) | (-1.33) | (-0.11) | (-0.16) | (2.06) |
|  |  |  |  |  |  |  |  |
| Citation, | 0.0139 | 0.0234 | 0.0276 | 0.00583 | 0.0282 | 0.0370 | 0.121^*^ |
| revealed x | (0.63) | (0.91) | (1.21) | (0.23) | (1.02) | (0.72) | (2.49) |
| value |  |  |  |  |  |  |  |
| Citation, not | 0.0347 | 0.0109 | 0.0244 | 0.00810 | 0.0160 | 0.0458 | 0.0792 |
| revealed x | (1.61) | (0.44) | (1.09) | (0.33) | (0.60) | (0.93) | (1.65) |
| value |  |  |  |  |  |  |  |
| Code, revealed | 0.0714^***^ | 0.0220 | 0.0367^*^ | 0.0438^*^ | 0.0533^**^ | 0.0660 | -0.0166 |
| x value | (4.42) | (1.17) | (2.19) | (2.37) | (2.64) | (1.82) | (-0.46) |
|  |  |  |  |  |  |  |  |
| Code, not | 0.0975^***^ | 0.0787^***^ | 0.0614^***^ | 0.0600^***^ | 0.0630^***^ | 0.116^***^ | 0.0464 |
| revealed x | (6.60) | (4.54) | (4.02) | (3.54) | (3.40) | (3.50) | (1.42) |
| value |  |  |  |  |  |  |  |
|  |  |  |  |  |  |  |  |
| Constant | 68.90^***^ | 65.75^***^ | 72.17^***^ | 74.81^***^ | 67.81^***^ | 65.26^***^ | 61.82^***^ |
|  | (53.23) | (43.64) | (53.64) | (50.46) | (41.72) | (21.66) | (21.34) |
| *N* (observations) | 9,498 | 9,006 | 9,334 | 8,712 | 8,840 | 4,381 | 4,466 |
| *N* (papers) | 4,223 | 4,136 | 4,194 | 4,081 | 4,086 | 2,716 | 2,745 |
| *R²* | 0.017 | 0.006 | 0.007 | 0.005 | 0.005 | 0.015 | 0.011 |

*Notes*. *t* statistics in parentheses; * *p*<0.05, ** *p*<0.01, *** *p*<0.001

Table A7. Fixed effects regression of quality assessments on presented numbers. Reviews and technical reports have been excluded.

| Variable | Coefficient |
| --- | --- |
| Citation, not revealed | -1.424 |
|  | (-0.90) |
|  |  |
| Code, revealed | -0.937 |
|  | (-0.57) |
|  |  |
| Code, not revealed | -1.648 |
|  | (-1.03) |
|  |  |
| Citation, revealed x value | 0.0152 |
|  | (0.67) |
|  |  |
| Citation, not revealed x value | 0.0333 |
|  | (1.51) |
|  |  |
| Code, revealed x value | 0.0759^***^ |
|  | (4.52) |
|  |  |
| Code, not revealed x value | 0.0944^***^ |
|  | (6.17) |
|  |  |
| Constant | 68.87^***^ |
|  | (51.71) |
| *N* (observations) | 8,999 |
| *N* (papers) | 4,031 |
| *R²* | 0.017 |

*Notes*. *t* statistics in parentheses; * *p*<0.05, ** *p*<0.01, *** *p*<0.001

Table A8. Fixed effects regression of quality assessments on presented numbers. Respondents have been excluded who searched for citation counts.

| Variable | Coefficient |
| --- | --- |
| Citation, not revealed | -1.952 |
|  | (-1.20) |
|  |  |
| Code, revealed | -1.369 |
|  | (-0.81) |
|  |  |
| Code, not revealed | -2.562 |
|  | (-1.58) |
|  |  |
| Citation, revealed x value | 0.00205 |
|  | (0.09) |
|  |  |
| Citation, not revealed x value | 0.0316 |
|  | (1.40) |
|  |  |
| Code, revealed x value | 0.0726^***^ |
|  | (4.29) |
|  |  |
| Code, not revealed x value | 0.103^***^ |
|  | (6.67) |
|  |  |
| Constant | 69.23^***^ |
|  | (51.13) |
| *N* (observations) | 8,721 |
| *N* (papers) | 4,027 |
| *R²* | 0.0197 |

*Notes*. *t* statistics in parentheses; * *p*<0.05, ** *p*<0.01, *** *p*<0.001

Table A9. Fixed effects regression of quality assessments on presented numbers. Papers with a large difference between presented citations and citations up to 2021 have been excluded.

| Variable | Coefficient |
| --- | --- |
| Citation, not revealed | -0.722 |
|  | (-0.37) |
|  |  |
| Code, revealed | 0.234 |
|  | (0.12) |
|  |  |
| Code, not revealed | -0.824 |
|  | (-0.44) |
|  |  |
| Citation, revealed x value | 0.0265 |
|  | (0.99) |
|  |  |
| Citation, not revealed x value | 0.0388 |
|  | (1.44) |
|  |  |
| Code, revealed x value | 0.0729^***^ |
|  | (4.39) |
|  |  |
| Code, not revealed x value | 0.0992^***^ |
|  | (6.54) |
|  |  |
| Constant | 67.81^***^ |
|  | (41.09) |
| *N* (observations) | 8,745 |
| *N* (papers) | 4,128 |
| *R²* | 0.0188 |

*Notes*. *t* statistics in parentheses; * *p*<0.05, ** *p*<0.01, *** *p*<0.001

Table A10. Fixed effects regression of quality assessments on presented numbers for different fields of citing papers.

| Variable | Natural sciences | Engineering& technology | Medical & health sciences | Social sciences |
| --- | --- | --- | --- | --- |
| Citation, not revealed | -1.982 | -6.091 | 3.748 | -5.746 |
|  | (-0.83) | (-1.06) | (0.99) | (-1.80) |
|  |  |  |  |  |
| Code, revealed | -2.460 | -11.85 | 2.676 | 2.476 |
|  | (-0.96) | (-1.90) | (0.72) | (0.75) |
|  |  |  |  |  |
| Code, not revealed | -2.302 | -9.587 | -0.828 | -2.064 |
|  | (-0.94) | (-1.62) | (-0.23) | (-0.64) |
|  |  |  |  |  |
| Citation, revealed x value | -0.00520 | -0.0661 | 0.0644 | 0.00605 |
|  | (-0.15) | (-0.74) | (1.23) | (0.13) |
|  |  |  |  |  |
| Citation, not revealed x value | 0.0393 | -0.0156 | -0.0281 | 0.102^*^ |
|  | (1.22) | (-0.17) | (-0.54) | (2.20) |
|  |  |  |  |  |
| Code, revealed x value | 0.0917^***^ | 0.123^*^ | 0.0287 | 0.0242 |
|  | (3.85) | (1.98) | (0.70) | (0.71) |
|  |  |  |  |  |
| Code, not revealed x value | 0.0904^***^ | 0.0315 | 0.140^***^ | 0.0621 |
|  | (4.23) | (0.49) | (3.96) | (1.93) |
|  |  |  |  |  |
| Constant | 69.86^***^ | 75.70^***^ | 65.27^***^ | 70.04^***^ |
|  | (33.35) | (14.74) | (21.44) | (26.96) |
| *N* (observations) | 5,118 | 1,395 | 2,355 | 1,996 |
| *N* (papers) | 2,632 | 1,057 | 1,477 | 844 |
| *R²* | 0.018 | 0.026 | 0.031 | 0.018 |

*Notes*. *t* statistics in parentheses; * *p*<0.05, ** *p*<0.01, *** *p*<0.001

Table A11. Fixed effects regression of quality assessments on presented numbers and knowledge of the cited paper.

| Variable | Coefficient |
| --- | --- |
| Citation, not revealed | -1.467 |
|  | (-0.98) |
|  |  |
| Code, revealed | -0.799 |
|  | (-0.51) |
|  |  |
| Code, not revealed | -1.635 |
|  | (-1.09) |
|  |  |
| Citation, revealed x value | 0.0348 |
|  | (0.95) |
|  |  |
| Citation, not revealed x value | 0.0336 |
|  | (0.94) |
|  |  |
| Code, revealed x value | 0.0661 |
|  | (1.85) |
|  |  |
| Code, not revealed x value | 0.0488 |
|  | (1.50) |
|  |  |
| Knowledge | -3.822^***^ |
|  | (-8.62) |
|  |  |
| Citation, revealed x value x knowledge | -0.00618 |
|  | (-0.66) |
|  |  |
| Citation, not revealed x value x knowledge | 0.00209 |
|  | (0.23) |
|  |  |
| Code, revealed x value x knowledge | 0.00329 |
|  | (0.34) |
|  |  |
| Code, not revealed x value x knowledge | 0.0143 |
|  | (1.52) |
|  |  |
| Constant | 80.79^***^ |
|  | (42.64) |
| *N* (observations) | 9,468 |
| *N* (papers) | 4,216 |
| *R²* | 0.0748 |

*Notes*. *t* statistics in parentheses; * *p*<0.05, ** *p*<0.01, *** *p*<0.001

Table A12. Fixed effects regression of quality assessments on presented numbers and proximity between respondents’ area of research and field of cited paper.

|  | (1) |
| --- | --- |
| Citation, not revealed | -1.032 |
|  | (-0.66) |
|  |  |
| Code, revealed | -0.643 |
|  | (-0.39) |
|  |  |
| Code, not revealed | -1.806 |
|  | (-1.15) |
|  |  |
| Citation, revealed x value | 0.0201 |
|  | (0.78) |
|  |  |
| Citation, not revealed x value | 0.0374 |
|  | (1.48) |
|  |  |
| Code, revealed x value | 0.0988^***^ |
|  | (4.55) |
|  |  |
| Code, not revealed x value | 0.0898^***^ |
|  | (4.56) |
|  |  |
| Proximity | 0.00938 |
|  | (0.57) |
|  |  |
| Citation, revealed x value x proximity | -0.000104 |
|  | (-0.30) |
|  |  |
| Citation, not revealed x value x proximity | -0.000157 |
|  | (-0.47) |
|  |  |
| Code, revealed x value x proximity | -0.000728^*^ |
|  | (-2.04) |
|  |  |
| Code, not revealed x value x proximity | 0.000202 |
|  | (0.60) |
|  |  |
| Constant | 68.42^***^ |
|  | (46.95) |
| *N* (observations) | 9,226 |
| *N* (papers) | 4,160 |
| *R²* | 0.0182 |

*Notes*. *t* statistics in parentheses; * *p*<0.05, ** *p*<0.01, *** *p*<0.001
